# Deformation-induced grain growth and twinning in nanocrystalline palladium thin films

**DOI:** 10.3762/bjnano.4.64

**Published:** 2013-09-24

**Authors:** Aaron Kobler, Jochen Lohmiller, Jonathan Schäfer, Michael Kerber, Anna Castrup, Ankush Kashiwar, Patric A Gruber, Karsten Albe, Horst Hahn, Christian Kübel

**Affiliations:** 1 Technische Universität Darmstadt (TUD), KIT-TUD Joint Research Laboratory Nanomaterials, 64287 Darmstadt, Germany; 2 Karlsruhe Institute of Technology (KIT), Institute of Nanotechnology (INT), 76021 Karlsruhe, Germany; 3 Karlsruhe Institute of Technology (KIT), Institute for Applied Materials (IAM), P.O. Box 3640, 76021 Karlsruhe, Germany; 4 Technische Universität Darmstadt (TUD), Petersenstr. 32, 64287 Darmstadt, Germany; 5 Department of Metallurgical and Materials Engineering, Visvesvaraya National Institute of Technology (VNIT), Nagpur 440 010, Maharashtra, India; 6 Department of Materials Engineering, Indian Institute of Science (IISc), Bangalore 560 012, Karnataka, India; 7 Karlsruhe Institute of Technology (KIT), Karlsruhe Nano Micro Facility (KNMF), 76021 Karlsruhe, Germany

**Keywords:** ACOM-TEM, deformation mechanism, nanostructured metals, tensile testing, XRD

## Abstract

The microstructure and mechanical properties of nanocrystalline Pd films prepared by magnetron sputtering have been investigated as a function of strain. The films were deposited onto polyimide substrates and tested in tensile mode. In order to follow the deformation processes in the material, several samples were strained to defined straining states, up to a maximum engineering strain of 10%, and prepared for post-mortem analysis. The nanocrystalline structure was investigated by quantitative automated crystal orientation mapping (ACOM) in a transmission electron microscope (TEM), identifying grain growth and twinning/detwinning resulting from dislocation activity as two of the mechanisms contributing to the macroscopic deformation. Depending on the initial twin density, the samples behaved differently. For low initial twin densities, an increasing twin density was found during straining. On the other hand, starting from a higher twin density, the twins were depleted with increasing strain. The findings from ACOM-TEM were confirmed by results from molecular dynamics (MD) simulations and from conventional and in-situ synchrotron X-ray diffraction (CXRD, SXRD) experiments.

## Introduction

Nanocrystalline (nc) metals and alloys exhibit different mechanical behavior compared to their coarse-grained counterparts [1]. They show extraordinary strength but often lack ductility and suffer from rapid strain localization [2]. Understanding the underlying deformation mechanisms operating in these structures is important, for example to guarantee the reliability of nc materials in next-generation micro- and nano-scale devices.

In nc metals, with grain sizes well below 100 nm, the conventional deformation mechanisms based on dislocation motion and multiplication, which govern deformation in coarse-grained metals, are increasingly limited by grain boundaries with decreasing grain size. It is believed that their low ductility is associated with this [3]. Although nc metals are under investigation for a number of years, there still is an ongoing debate on the deformation mechanisms active in these materials. Discussed are grain boundary sliding, grain rotation, emission and annihilation of dislocations at grain boundaries, intragranular dislocation glide resulting in twinning/detwinning processes, stress-driven grain boundary migration and the formation of shear bands [4–7].

When studying the mechanical properties of nc metals and the associated deformation mechanisms, it is important to consider the preparation technique for the corresponding bulk nc metal. Bulk nc metals are typically produced by severe plastic deformation [8–11], inert gas condensation [4,12] or electrochemical deposition [13]. The different approaches result in significant differences in dislocation and twin density, porosity and impurity levels of the nc metals, where, e.g., pores and impurities may pin grain boundaries [14–15]. Furthermore, the different production techniques may also lead to different grain boundary structures with varying free volume and defect structure [4,16–17]. All these factors will affect the mechanical behavior of the material, making it difficult to determine and compare the inherent properties. As an alternative approach to prepare dense and very pure nc metals, we employed interrupted magnetron sputtering of thin metallic films [3,18–19]. The drawback of this approach is that mechanical testing and handling of the films is difficult. However, this can be improved by sputtering onto compliant polyimide substrates to stabilize the specimens and to avoid strain localization during tensile testing [20–22]. Such samples were successfully used in the present work.

The goal of the present work is to investigate the deformation processes active in ncPd films deposited by magnetron sputtering onto compliant substrates. The microstructural analysis is mainly performed by quantitative automated crystal orientation mapping TEM (ACOM-TEM) [23–24] and supplemented with grain size measurement using dark-field TEM (DF-TEM) and conventional X-ray diffraction (CXRD) after straining to different deformation levels. The evaluation presented in this paper concentrates on grain growth and twinning due to straining. The findings from the ex-situ investigations were compared to results obtained during in-situ deformation experiments using synchrotron X-ray diffraction (SXRD) on equivalent samples. Furthermore, the experimental results for the twin density evolution are qualitatively supported by molecular dynamics (MD) simulations.

## Results

### Microstructural characterization of the as deposited Pd films

The sputtering parameters were chosen to minimize residual stress and to obtain dense and high purity ncPd films [18]. Although the sputter parameters were chosen to be the same for each sample set, small fluctuations in the pressure have led to sample sets with slightly different residual stresses and different initial twin densities. Table 1 gives an overview of the samples analyzed.

**Table 1:** Overview of the structural properties of the three ncPd sample sets analyzed using different techniques before straining.

sample sets			ncPd 1	ncPd 2	ncPd 3

crystallite size [nm]	plane-view	ACOM-TEM	23 ± 13	24 ± 15	—
		in-situ SXRD	—	—	17

	growth direction	ACOM-TEM	34 ± 35	43 ± 23	—
		CXRD (111) peak	68	61	—

grain size [nm]	plane-view	ACOM-TEM	36 ± 19	34 ± 22	—
		DF-TEM	—	49	—
		in-situ SXRD	—	—	38

twin boundaries/ per grain		ACOM-TEM	1.3	1.1	—
		DF-TEM	—	0.005	—
		in-situ SXRD	—	—	1.2^a^

CXRD residual stress [MPa]			−47 ± 25	16 ± 25	—

CXRD micro strain			0.004	0.004	—


^a^Determined only by comparing grain size and crystallite size, assuming that the difference arises only from twinning. For details see section ‘Twin density evolution’.

**Figure 1:**
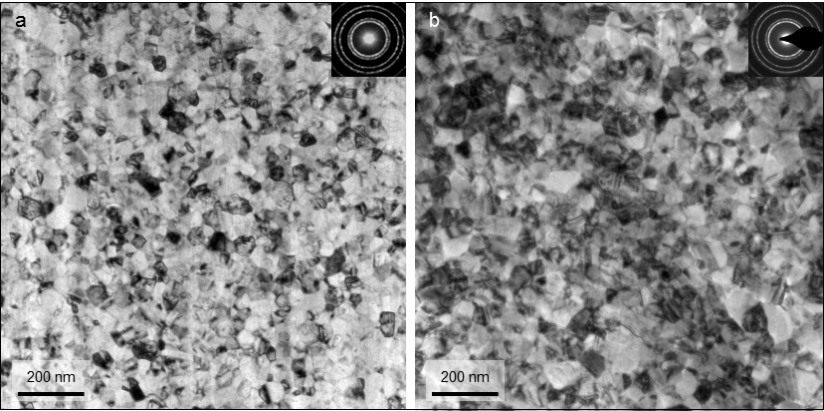
BF-TEM images of the initial microstructure of ncPd 1 (a) and ncPd 2 (b) with the corresponding selected electron diffraction pattern (SAED) as insets.

**Figure 2:**
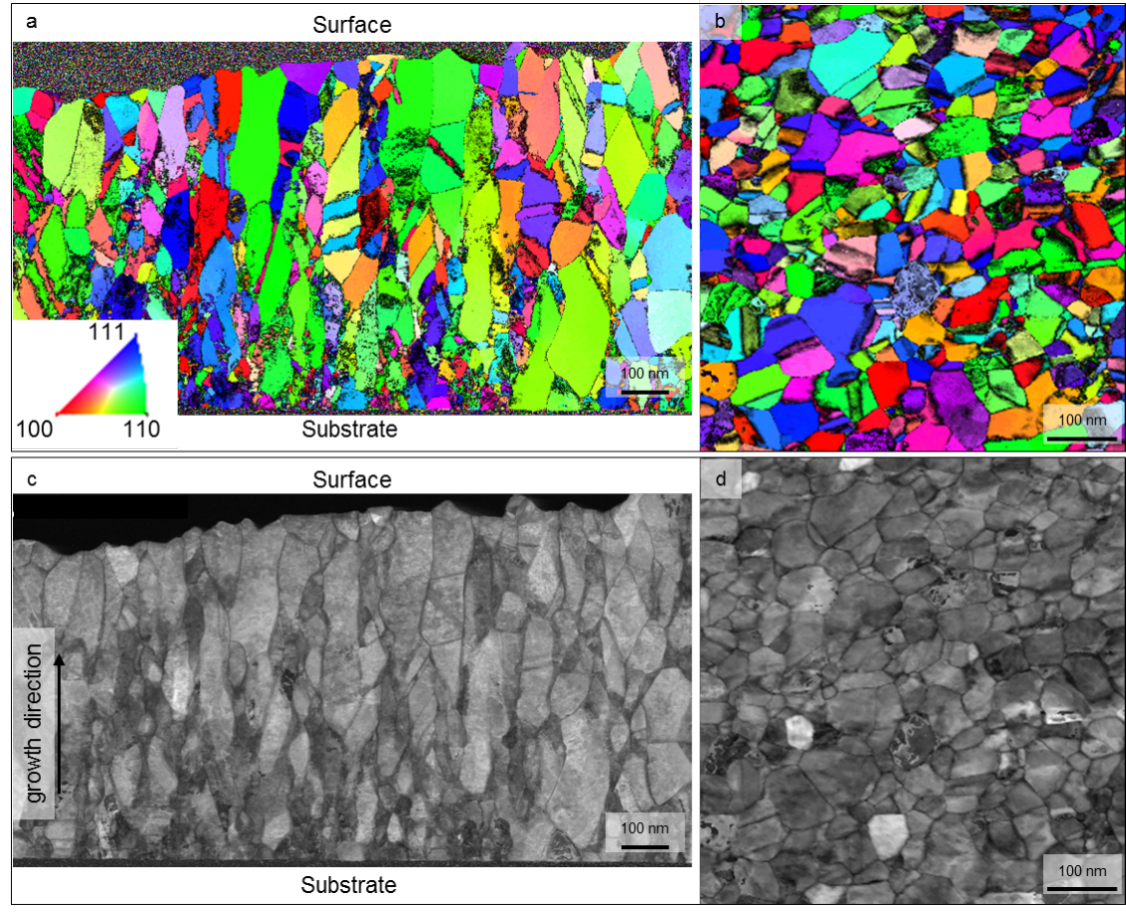
Orientation maps overlaid with reliability derived from ACOM-TEM of the as deposited sample in a) cross section and b) plane view (insets shows the color code for the orientation maps), c) and d) are the corresponding ACOM-TEM cross correlation index maps of the cross section and plane view, respectively.

BF-TEM images of the as-deposited sputtered Pd films exhibit a comparable microstructure for sample ncPd 1 and ncPd 2 (Figure 1). Figure 2 displays the microstructure as revealed by ACOM-TEM in plane-view and cross section. It shows an elongated columnar grain structure in growth direction compared to the isotropic structure in plane-view typical for sputter-deposited thin films.

**Figure 3:**
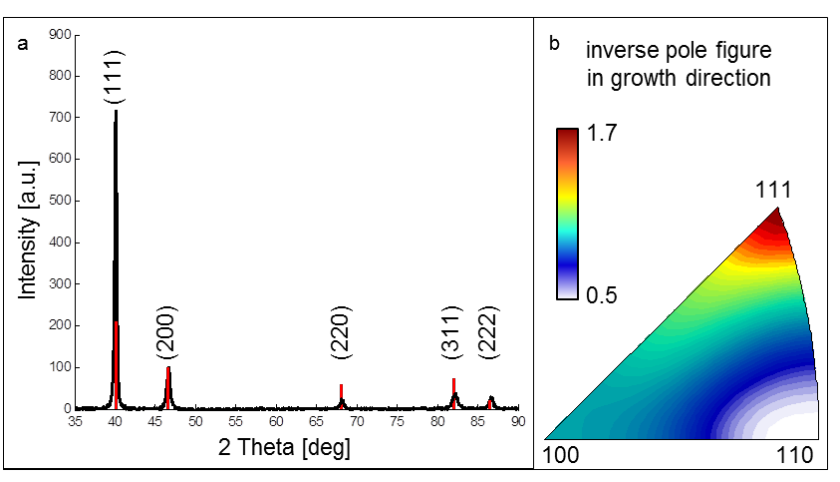
Orientation analysis of the as prepared sputtered ncPd film. a) X-ray diffraction pattern of the as deposited sample (black) (ncPd 2) overlaid with the isotropic Pd intensity profile (red) (normalized to the (200) peak intensity). b) Orientation density function (inverse pole figure) determined by ACOM-TEM in growth direction (ncPd 1).

CXRD analysis revealed an increased ratio between the XRD intensity of the (111) peak and the (200) peak of 9.8:1 for sample ncPd 1 and 7:1 for sample ncPd 2 compared to 2.1:1 for an isotropic palladium powder (Figure 3a). This indicates a <111> texture component in growth direction, which was also observed in the ACOM orientation density function in growth direction for both ncPd sample sets (Figure 3b).

### Grain size evolution

**Figure 4:**
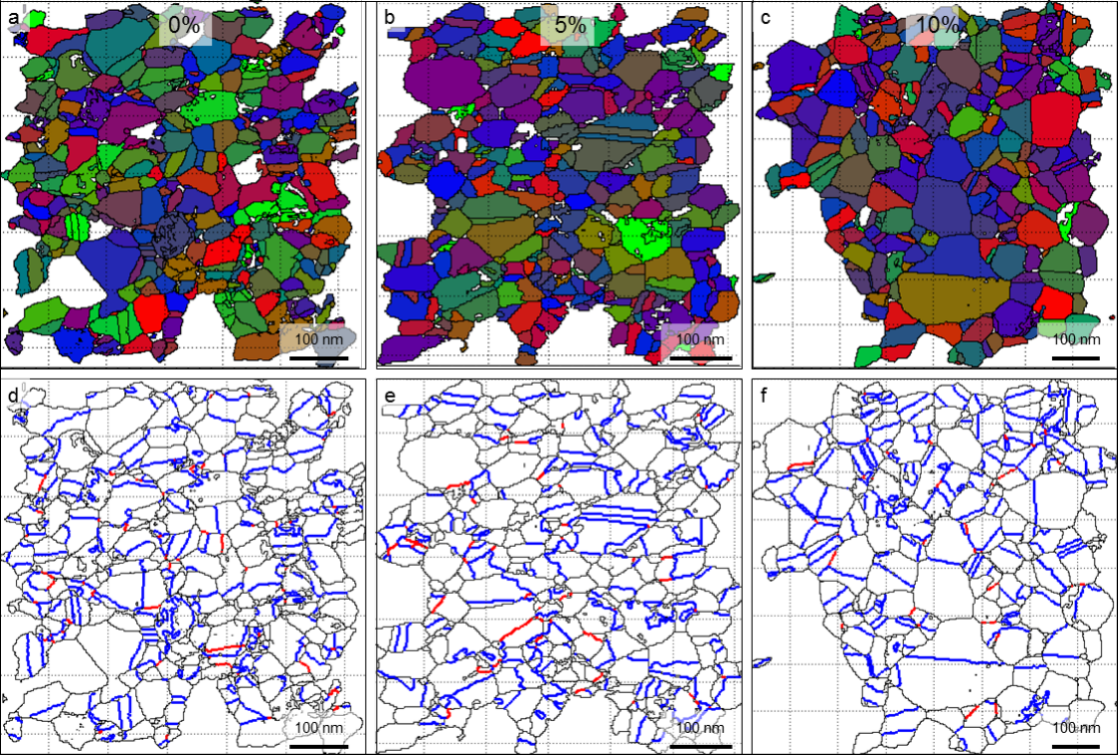
Crystallites recognized by ACOM-TEM for the as deposited sample and samples deformed to 5% and 10% strain (a–c). d–f) Crystallite boundaries (black), twin boundaries (CSL Σ3) (blue) and CSL Σ9 boundaries (red).

**Figure 5:**
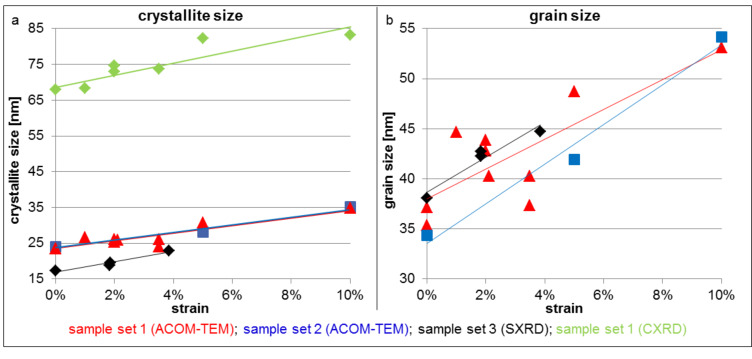
a) Crystallite and b) grain size as a function of strain based on ACOM-TEM (equivalent in-plane diameter) (ncPd 1: red, ncPd 2: blue), CXRD (out-of-plane grain size) (ncPd 1: green) and in-situ SXRD (area weighted in-plane diameter) (ncPd 3: black) analysis.

Different characterization techniques result in slightly different absolute values for the grain size, but the trends observed during straining for each sample set are systematic and comparable for all investigation techniques used. The microstructural evolution during deformation of sample sets ncPd 1 and ncPd 2 was investigated by ACOM-TEM. Orientation and crystallite boundary maps corresponding to 0%, 5% and 10% strain are displayed in Figure 4. The grain size increases, with no noticeable evolution of a bimodal size distribution and no significant preferential growth direction is observed in plane-view. In Figure 5a, a quantitative analysis of the crystallite size as a function of strain is given for all sample sets and analysis methods. Independent of sample set and method, an increase in in-plane crystallite size with increasing strain was observed, leading to an increase in crystallite diameter of ca. 45% at 10% strain for sample sets ncPd 1 and ncPd 2. This trend was also confirmed by in-situ SXRD and sample set ncPd 3. On the other hand, the CXRD crystallite size, representing the crystallite size perpendicular to the film surface, only increased by ca. 22%. Comparing the out-of-plane grain size evolution observed by CXRD and the in-plane ACOM-TEM analysis suggests a non-isotropic crystallite growth within the plane of the thin film and perpendicular to it with a ratio of around 2. This observed difference in crystallite growth is probably the result of stress coupled grain boundary motion, with a higher stress within the plain of the sample compared to the perpendicular direction. However, as we compare the crystallite size only we cannot exclude differences in the twin boundary motion behavior in both directions, which could contribute further to the crystallite size changes. Further, the columnar structures of the metal films may contribute to the observed difference in crystallite growth within the film and perpendicular to it.

When analyzing the grain size instead of the crystallite size, the effect of twinning is removed from the grain growth (Figure 5b). In the case of sample ncPd 1, this reveals a similar trend for the grain growth as observed for the crystallite growth, but a significantly more pronounced grain growth of about 58% for sample ncPd 2. The difference is due to the different twinning behavior of both sample sets, described in the next section.

### Twin density evolution

**Figure 6:**
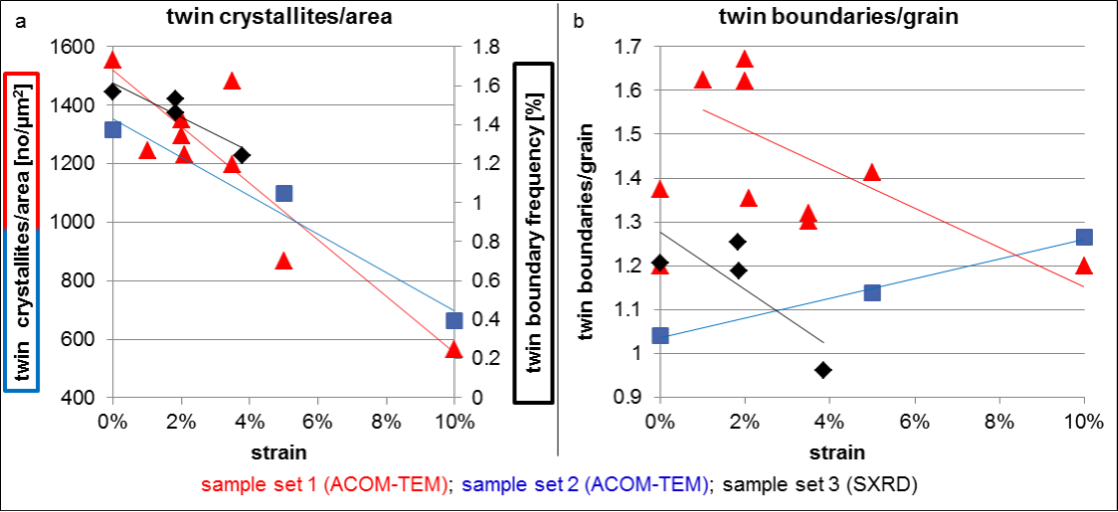
a) Twin crystallites/area as a function of strain based on the ACOM-TEM analysis (ncPd 1: red, ncPd 2: blue) and twin boundary frequency (*f*_twin_) as a function of strain (ncPd 3: black) derived from in-situ SXRD data. b) Twin boundaries/grain as a function of strain based on the ACOM-TEM (ncPd 1: red, ncPd 2: blue) and in-situ SXRD analysis (ncPd 3: black).

Twinning was analyzed using ACOM-TEM, in-situ SXRD and MD simulations. In order to understand the twin density evolution during straining, the measurement metric is very important. When analyzing the twin density as twins per area, all sample sets exhibit a decrease in twin density during straining up to 10% total strain, namely by 82% for sample ncPd 1 and by 50% for sample ncPd 2, respectively (Figure 6a). However, this metric is mixing grain size changes and twin density evolution. To separate the twin density evolution from grain growth, we evaluated the number of twin boundaries per grain (Figure 6b). With this metric, the two sample sets showed a significantly different behavior. While the twin boundaries per grain decreased with increasing strain for sample ncPd 1 by 26%, it increased for sample ncPd 2 by 22%. The twin boundaries per grain determined for the initial structure of sample ncPd 1 do not seem to fit to the overall trend observed during straining. However, contrary to all the other samples, the analysis for the initial structure of sample ncPd 1 was performed close to the end of the sample stripe which was used for clamping of the sample during mechanical testing. Although the sputtering was conducted with substrate rotation, there might be a slight difference in the microstructure between the middle of the sample and the edge of the test samples. Therefore, the grain size/twin density of the initial sample ncPd 1 is not considered for the analysis. The number of twin boundaries per grain in both sample sets after 10% straining are quite similar, despite the different initial levels, suggesting that the twin boundary density might converge to an equilibrium value during deformation.

The trend of decreasing twin density per area seen by ACOM-TEM was also observed for sample ncPd 3 using in-situ SXRD and subsequent X-ray line profile analysis (Figure 6a). However, the metric accessible from XRD is the twin boundary frequency. It describes the mean probability (in percent) that a lattice plane within the ensemble of (111) planes is a twin plane and is based on a purely statistical analysis established for single crystals with randomly distributed twin boundaries. For example, here the initial twin boundary frequency of *f*_twin_(0) = 1.57% means that on average every 67th (111) lattice plane is a twin plane, corresponding to an average spacing between two twin boundaries of about 15 nm for (111) twins in Pd. It is known that the twin boundary frequency obtained by XRD tends to overestimate the number of twin boundaries, especially if they are located close to the center of a grain [25–27] which is preferentially the case here for the growth twins in the as-deposited Pd film (Figure 4d). Consequently, the twin spacing given above is difficult to directly compare with the ACOM-TEM analysis. As an alternative approach for a direct comparison between SXRD and ACOM-TEM analysis, we, therefore, propose the ratio of grain and crystallite size to estimate the number of twin boundaries per grain. It can be approximated as

[1]
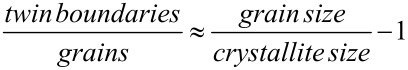


leading to 1.2 twin boundaries/grain based on the in-situ SXRD analysis. This is in good agreement with the corresponding values obtained via ACOM-TEM. The twin boundary frequency obtained by SXRD decreases from *f*_twin_(0) = 1.57% to *f*_twin_(3.8) = 1.24% upon straining to 3.8% (Figure 6a) and the resulting values for the twin boundaries/grain are in agreement with the trend observed by ACOM-TEM (Figure 6b).

**Figure 7:**
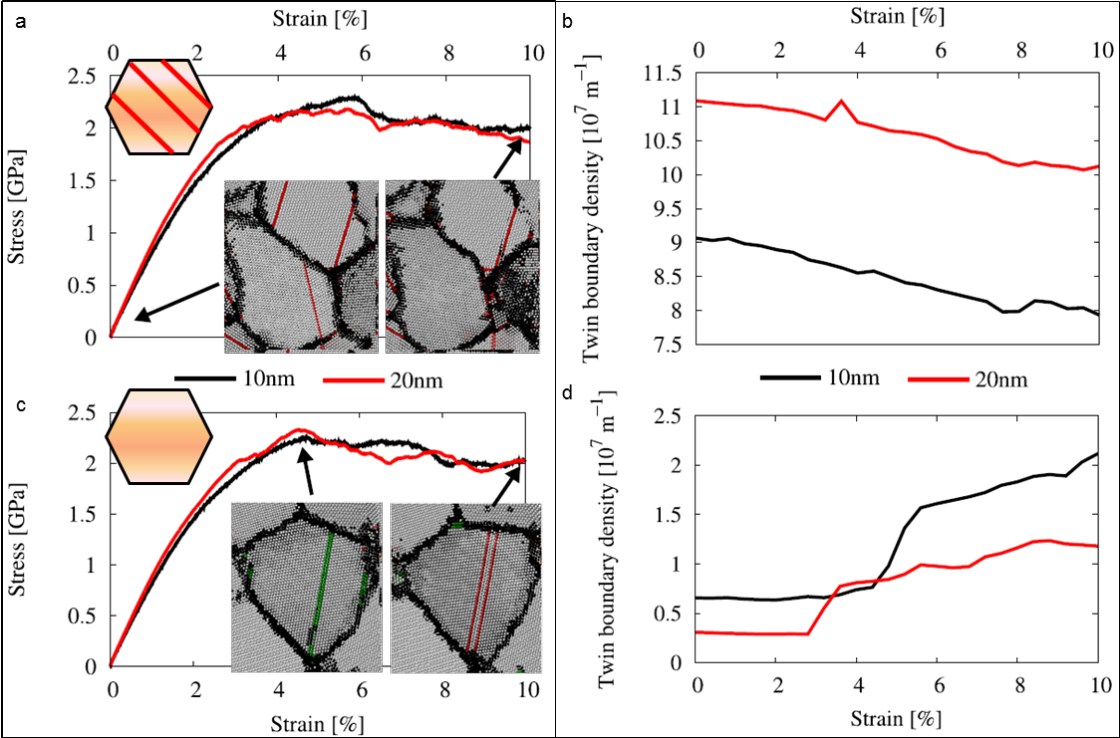
Stress strain behavior and evolution of twin boundary density as a function of strain for grain sizes of 10 and 20 nm based on MD simulations. Two different initial microstructures are compared: a) and b) show the data for structures initially loaded with growth twins (50% of the grains are loaded with twin boundaries) and c) and d) without twin boundaries present prior to straining. The snapshots show representative slices through the microstructure at different stages of the deformation. Atoms in the grain boundary are highlighted in black and atoms located in a stacking fault or a twin boundary are highlighted in green and red.

In addition to the experimental investigations, MD simulations were conducted to elucidate the twinning evolution during straining, starting from different initial configurations. Figure 7 shows the stress–strain behavior and the corresponding evolution of the twin boundary density during straining for 4 different cases with varying initial twin density and different grain size. Starting from a high initial density of twin boundaries (e.g., growth twins), the twin density decreases with increasing strain (Figure 7a,b). In the slices through representative grains at different stages of the deformation, it is shown that twin boundaries (highlighted in red) disappear from the microstructure. This occurs by nucleation of partial dislocations at twin boundaries and their successive motion (not shown). In contrast, samples with an initially defect free grain interior showed an increase of the twin boundary density during straining (Figure 7c,d). Twins nucleate at the grain boundaries under an applied stress by emission of partial dislocations, which lead to stacking faults and eventually twin embryos. These trends are independent of the studied grain sizes.

## Discussion

Grain growth of magnetron sputtered ncPd films was evaluated by comparing analysis of ex-situ and in-situ tensile tests. For ex-situ investigations, individual samples of two sample sets (ncPd 1 and ncPd 2) were strained to defined states and investigated using TEM and CXRD. A third sample (ncPd 3) was analyzed by in-situ SXRD during cyclic loading. The results from SXRD given here represent unloaded sample states to ensure comparability with the ex-situ results. Both approaches provide results in good qualitative agreement. In all three sample sets investigated, grain growth as a result of increasing strain was evident. With both ACOM-TEM and SXRD, we showed that not only the crystallite size increases with increasing strain but also the grain size. This indicates that the applied stress was accommodated by grain boundary motion in addition to the observed twinning/detwinning. The observed grain growth is in line with a recent in-situ XRD study on the deformation behavior of similar sputter-deposited ncPd thin films [28], but is in contrast to measurements performed on electron beam evaporated Pd samples where no grain growth was reported up to 4% strain [29]. However, the same authors also compared the microstructure of electron-beam evaporated and magnetron sputtered samples [30] and found significantly higher initial twin densities in electron-beam evaporated Pd films. This difference in microstructure along with slightly different impurity levels may alter the grain boundary mobility and may explain the different grain growth behavior of sputtered and electron-beam evaporated Pd films. The comparison between in-situ and ex-situ analysis shows that ex-situ analysis of different straining states is possible with a quantitative evaluation of grain growth by ACOM-TEM as the nc structure is sufficiently homogeneous in case of the magnetron sputter deposited ncPd films. Grain growth as a result of aging at room temperature can be excluded to contribute to our evaluation based on the XRD analysis of undeformed parts of the tensile samples after 12 weeks, which did not exhibit a significant change in grain size.

Twinning is a possible mode for plastic deformation that is mainly observed in fcc metals with low stacking fault energy, but also in fcc metals with high stacking fault energy if deformed under extreme conditions, like cryogenic temperatures, shock loading, or large strains [13]. The high stacking fault energy of Pd (169 mJ/m^2^) [31] should lead to a low number of twinned grains. However, ACOM-TEM analysis reveals that the investigated system inherently contains 1.3 (ncPd 1) / 1.1 (ncPd 2) growth twin boundaries per grain. This has not been evident from classical DF-TEM (see Supporting Information File 1).

The two sample sets examined here in detail, show different initial microstructures: the sample grown with slight compressive residual stress (Table 1) exhibits a higher twin density compared to the sample grown with slight tensile stress. The higher amount of growth twins did not affect the flow stress in our experiments (not shown here). This was also confirmed by the MD-simulations, which showed no significant difference in the stress strain behavior after introducing twin boundaries to 50% of the grains (Figure 7a,c). However, simulations by Stukowski et al. showed a softening effect due to twin boundaries in Pd [32]. The simulations of Stukowski et al. and the simulations presented here differ in two ways. In the study by Stukowski et al., all grains were loaded with twins and a symmetrical grain shape was chosen, which suppresses grain boundary mediated processes. If all grains in our samples are loaded with twin boundaries, we also see a softening effect. Experiments and simulations further show, that the softening of Pd due to twin boundaries differs from observations made for nano-twinned Cu, where twin boundaries led to strengthening [32–34]. The difference between Cu and Pd can be explained by the preferred nucleation site of partial dislocations: In case of nano-twinned Cu, partial dislocations nucleate from the grain boundaries onto planes with the highest Schmid-factor (the highest resolved shear stress). Here, randomly oriented twin boundaries can act as barriers to dislocation motion. In nano-twinned Pd, however, partial dislocations nucleate preferentially at twin boundaries. The twin boundaries then act as nucleation site and do not hinder successive dislocation motion in the twinning plane [32]. The preferential nucleation of partial dislocations at twin boundaries is consistent, with the evolution of the twin boundary density in the present work, where a high initial density leads to an annihilation of twin boundaries during straining.

**Figure 8:**
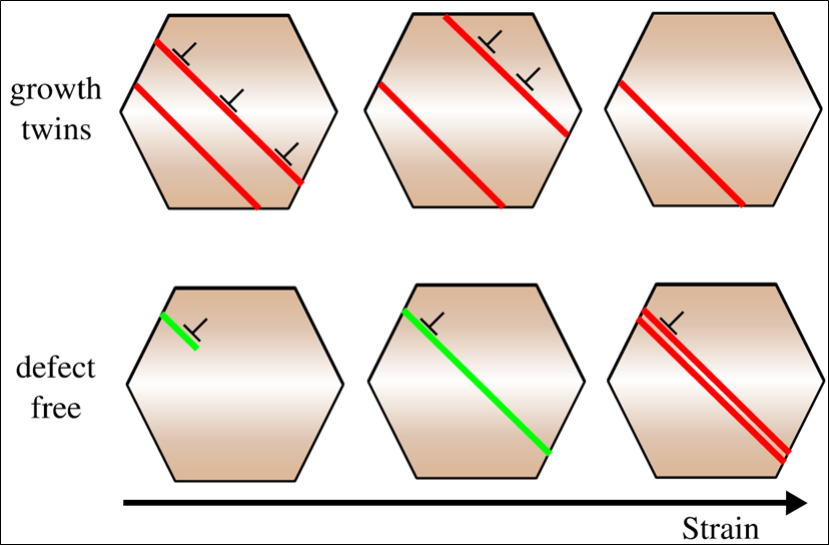
Model of the deformation pathways: If growth twins are initially present, partial dislocations nucleate at twin boundaries and lead to twin boundary migration out of the grain. If the grains are initially defect free, partial dislocations nucleate into a defect free grain and can form stacking faults and twinning faults by successive nucleation of partial dislocations.

The present experiments and simulations have shown that twinning/detwinning as a result of partial dislocation activity contributes to the accommodation of the induced strain. Figure 8 illustrates two competing processes taking place during deformation, depending on the initial twin boundary density. In case of a high initial growth twin density, partial dislocations nucleate preferentially at the twin boundaries and lead to movement and thus a reduction of twin boundaries [35]. If no twin boundaries are present, partial dislocations need to nucleate from the grain boundaries into defect free grains and may form stacking faults and subsequently individual twins by successive motion of partial dislocations [36]. The initial nucleation is controlled by the Schmid factor. As soon as stacking faults or twins are formed, the tendency is identical to the twinned case. Presumably this competition between annihilation and nucleation of twin boundaries consequently leads to a steady state twin density during straining, which is seen experimentally for the twin density of samples ncPd 1 and ncPd 2 approaching similar values at 10% strain. In the simulations, where the initial twin density difference was significantly higher, this steady state was not reached within 10% strain. Twinning and Detwinning have been reported in several experimental studies. Twinning has been reported for Cu [37] with a low stacking fault energy, for Al with a high stacking fault energy [38] and for the compound material NiFe [39]. Detwinning on the other side has been also reported for Cu [40], Al [41] and NiFe [42]. It has been proposed that twinning and detwinning occur at the same time with one process prevailing over the other depending on grain size [43]. In our measurements we could not confirm a grain size effect for the competing mechanisms as both investigated sample sets showed comparable grain sizes in the initial state, but a dependence on the initial twin density is evident.

The present simulation shows that dislocation plasticity is active and as a consequence twinning/detwinning processes take place during deformation. Carlton et al. estimated the dislocations required for purely dislocation based plasticity by

[2]
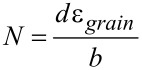


with *d* the grain size, ε_grain_ the strain in the grain (here considered to be 4%) and *b* the burgers vector, here considered for the slip system {111} and <110>, resulting in an average of 6.1 dislocations per grain [44]. On the experimental side, Lohmiller et al. showed for comparable samples that the dislocation density begins to increase in the micro plastic regime starting from 0.3–0.4% applied strain [28,45]. A dislocation density of ρ = 3.5 · 10^−3^ nm^−2^ was reported at 0% strain and 6 · 10^−3^ nm^−2^ at 4% strain. This corresponds to an average dislocation spacing of about 17 nm (0%) and 13 nm (4%), or about 2 (0%) and 3 (4%) dislocations per grain for a grain size of 35 nm (0%) and 41 nm (4%). This dislocation activity can explain the twin boundary mobility leading to the changes in twin density seen by ACOM-TEM. However, Carlton’s estimation for the dislocations needed for a purely dislocation based deformation is noticeably higher compared to the experimentally estimated dislocation density. The difference suggests that grain boundary mediated deformation mechanisms have to be active to compensate for the remaining strain that does not come from dislocation/twin activity. Lohmiller et al. has already reported other mechanisms, such as GB shear and slip, as well as GB migration resulting in grain growth [28,45].

## Conclusion

NcPd thin films with a grain size of about 35 nm (plane view) were sputter deposited onto flexible polyimide substrates. The samples were tensile tested ex-situ up to defined maximum strains and the resulting microstructure as a function of imposed strain was investigated using ACOM-TEM and CXRD after deformation. Two sets of sputtered samples were directly compared by ACOM-TEM. In addition, the results were confirmed with in-situ SXRD and with observations from MD simulations. The conclusions can be summarized as follows:

1. ACOM-TEM revealed a notable amount of growth twins present in the sputtered samples. 64–75% of the grains in the as prepared microstructure contain about 1–2 twins, with the absolute value depending on minute difference in the sputtering conditions.
2. Grain growth as a result of stress coupled grain boundary motion was identified as one of the deformation mechanism. The grain growth observed within the plane of the film was about a factor of 2 higher compared to the grain growth along the thickness of the film.
3. Partial dislocation activity leading to twinning/detwinning was identified as a second deformation mechanism. Samples with a high density of growth twins exhibit detwinning with increasing strain, whereas samples with a low density of growth twins react with twinning to increasing strain in qualitative agreement with MD simulations.
4. The main findings by ACOM-TEM on grain growth and twin density evolution were confirmed by careful in-situ SXRD analysis.

## Experimental

### Sample preparation

Palladium thin films were deposited by radio frequency (RF) magnetron sputtering using 2’’ diameter planar targets with 99.95% purity. Three sets of pure Pd samples were prepared with constant sputtering power of 60 W at a working gas pressure (Ar) of 0.005 mbar at room temperature. Pd films of 1 µm nominal thickness were grown in 100 steps of 10 nm film thickness, each interrupted for 10 s using a fast rotational shutter in front of the Pd-target. Polyimide Kapton E (DuPont) sheets with a thickness of 50 and 25 µm were used as substrate. Prior to film deposition, the substrates were cut into strips of 30 mm length and 5 mm width using a razor blade, cleaned in an ultrasonic acetone bath for 15 min, and rinsed with isopropanol on a resist spinner. This cleaning procedure, developed by Lohmiller et al. [20], provides enhanced adhesive support of the thin metallic film preventing strain localization and film delamination, allowing for a homogeneous deformation up to very high total strain. Directly after rinsing, the samples were clamped onto special sample holders and transferred into ultrahigh vacuum of 2.0 · 10^−8^ mbar within the sputtering chamber.

#### Mechanical testing and microstructural characterization

Ex-situ uniaxial tensile tests were carried out at room temperature using a dedicated tensile stage for miniature specimens at a strain rate of 5 · 10^−5^ s^−1^. The tensile tester was equipped with a special thin film sample clamping to avoid sample deformation during mounting of the delicate thin films. The elongation was measured using a laser extensometer (P-50, Fiedler Optoelectronics) to read paper marks applied to the backside of the polyimide substrate. The individual samples were pulled to maximum elongations of 1%, 2%, 3.5%, 5% and 10.0% engineering strain and relaxed for post-mortem TEM and CXRD analysis.

Specimens for post-mortem TEM analysis were prepared either by focused ion beam (FIB) using a FEI Strata 400S DualBeam at 5 kV and 8 pA beam current for final polishing (sample ncPd 1) or by mechanical dimpling and Argon ion milling from the polyimide side at 2.5 kV in a PIPS (Gatan) (sample ncPd 2). FIB prepared samples have the advantage that the loading direction can be tracked down to the TEM sample and the user can define the height inside the metal film for plane-view preparation. The later has an influence on the observed microstructure as the crystallite size shrinks towards the substrate [18]. Therefore, plane-view FIB samples shown here were always prepared close to the surface, typically from the middle of the straining section.

ACOM-TEM analysis was performed using a FEI Tecnai F20 ST operated at 200 kV in micro-probe (µp) STEM mode with spot size 8, camera length 100 mm, condenser aperture 30 µm, gun lens 6 and extraction voltage 4.5 kV, resulting in a spot size of about 1 nm and a semi convergence angle of 1.4 mrad. An ASTAR system (Nanomegas) was used for ACOM diffraction data acquisition. Data processing consisted of the following steps [23]:

1. Pixel filtering of the orientation maps with a median filter of the combined Euler angles.
2. Grain recognition in the orientation maps with an expanded Mtex version 3.3.1 [46]. The chosen disorientation between neighbors for the recognition is 3°.
3. Grain filtering: Removal of grains that contain less than 50% pixel having a combined reliability greater than 0.15 and index greater than 20.
4. Crystallite filtering: Removal of crystallites with an equivalent diameter <8 nm (ncPd 1) <10 nm (ncPd 2).
5. No re-filling of any of the removed pixels was performed.

Independent of the exact filter settings used in steps 2–4, the trends revealed in this study remain the same. Only slight changes in the absolute values result from using different filter settings. The general trends have also been confirmed using the TEAM^TM^ (EDAX) software as a well-established analysis package. The crystallite and grain sizes given for the ACOM results are the number-averaged equivalent diameters of the crystallites/grains. Only the histograms in Figure 4 show area weighted grain sizes. Here, a crystallite is defined as the smallest uniform crystallographic unit based on the disorientation to its neighbors. If a crystallite is separated from a neighbor by a twin boundary (recognized by the CSL Σ3 condition within the Brandon criteria) each of them are called twin crystallites. Grains can be single crystallites or consist of one or more twins. The twin density is defined as twin boundaries per grain.

BF/DF-TEM analysis was performed using an image corrected FEI Titan 80-300 equipped with a Gatan US1000 slow scan CCD camera operated at 300 kV. DF-TEM images were acquired with a 10 µm objective aperture at a scattering angle around 0.6°. Crystallite sizes were evaluated manually measuring at least 200 crystallites in different orientations for each straining level. The observed twin density is calculated as twin boundaries per grain detected by human inspection of the DF-TEM images.

Conventional XRD measurements were performed in Bragg–Brentano geometry using a Bruker D 8 Discover diffractometer to determine the crystallite size and residual stress. The measured profiles were evaluated in MATLAB using a home-built script. Peak analysis was performed based on the Single Line Method [47–48]. The crystallite size was determined from the broadening of the (111) peak. The (200) reflection was also analyzed showing a considerably smaller crystallite size compared to the (111) peak analysis. However, as the (200) peak has a considerable lower signal-to-noise ratio, in the present work only the crystallite size determined from the (111) reflection is given. The residual stress was investigated via the sin^2^Ψ method using the (311) and (331) peaks.

Additionally, similar samples were used for in-situ SXRD deformation experiments at the Material Science Beamline of the Swiss Light Source [28,45]. These samples were also subjected to tensile deformation at room temperature at a similar strain rate. In these experiments the samples were loaded in two cycles, first to a maximum elongation of 2% and subsequent unloading, followed by a second cycle to 3.8% and unloading. X-ray diffraction profiles were recorded continuously during deformation using 17.5 keV synchrotron radiation. For comparison with the ex-situ investigations in this work, only the results from the initial state, after the first cycle and at the end of the second cycle were used here (sample ncPd 3). The evaluation of the X-ray profiles was done using CMWP-fit [49] to determine the parameters of the grain-size distribution as well as the twin frequencies. The contribution of twinning in the evaluation of the X-ray data is a diffraction-order dependent part of the size broadening. If one does not consider the effect of twinning in the model, the broadening due to twinning manifests itself in a smaller CSD-size as the twin-faults are then treated as boundaries breaking the coherent scattering. According to the definition given earlier this is exactly the case referred to as the crystallite size. If twinning is considered in the model for the data evaluation, the size effect is reduced as the twin fault is considered as a special defect within a bigger volume. In this situation the size determined from the SXRD evaluation gives the grain size as defined in the ACOM section. More details on this experiment and the analysis can be found in references [28,45,50].

#### Simulations

MD simulations were carried out assuming pure ncPd as described by an embedded atom method potential [51]. For all simulations, the LAMMPS MD code [52] was used. The input structures were constructed following the Voronoi tessellation method [53], with an average grain size of 10 and 20 nm consisting of 64 and 36 grains, respectively. After initial relaxation at 300 K, in one sample of each grain size, 50% of the grains were loaded with twin boundaries with an average spacing of 4 nm. All samples were then annealed for 1 ns at 500 K to minimize the enthalpy. Successive uniaxial straining simulations were carried out with a strain rate of 10^8^ s^−1^. The evolution of defects during straining was extracted, using a dislocation extraction algorithm [54], which allows to identify the atoms in twin boundaries. The twin boundary density as a function of strain was computed as the atomic area of all atoms inside a twin boundary normalized by the total volume. Analysis and visualization was carried out using DXA [54] and OVITO [55].

## Supporting Information

File 1 Comparison between ACOM- and DF-TEM evaluation of grain growth and twin activity.
