# Supplementary material for: Deformation-induced grain growth and twinning in nanocrystalline palladium thin films
Source: Beilstein J Nanotechnol. 2013 Sep 24;4:554–66. doi: 10.3762/bjnano.4.64 (PMC3817795; doi:10.3762/bjnano.4.64)
Supplement: File 1 — Comparison between ACOM- and DF-TEM evaluation of grain growth and twin activity. [file Beilstein_J_Nanotechnol-04-554-s001.pdf]

Supporting Information

for

Deformation-induced grain growth and twinning in nanocrystalline palladium thin films

Aaron Kobler*[1,2], Jochen Lohmiller[3], Jonathan Schäfer[4], Michael Kerber[2], Anna Castrup[1,2], Ankush Kashiwar[5,6], Patric A. Gruber[3], Karsten Albe[4], Horst Hahn[1,2] and Christian Kübel*[2,7,§]

Address: [1]Technische Universität Darmstadt (TUD), KIT-TUD Joint Research Laboratory Nanomaterials, 64287 Darmstadt, Germany, [2]Karlsruhe Institute of Technology (KIT), Institute of Nanotechnology (INT), 76021 Karlsruhe, Germany, [3]Karlsruhe Institute of Technology (KIT), Institute for Applied Materials (IAM), P.O. Box 3640, 76021 Karlsruhe, Germany, [4]Technische Universität Darmstadt (TUD), Petersenstr. 32, 64287 Darmstadt, Germany, [5]Department of Metallurgical and Materials Engineering, Visvesvaraya National Institute of Technology (VNIT), Nagpur 440 010, Maharashtra, India, [6]Department of Materials Engineering, Indian Institute of Science (IISc), Bangalore 560 012, Karnataka, India and [7]Karlsruhe Institute of Technology (KIT), Karlsruhe Nano Micro Facility (KNMF), 76021 Karlsruhe, Germany

Email: Aaron Kobler - aaron.kobler@kit.edu, Christian Kübel - christian.kuebel@kit.edu

* Corresponding author

§ Hermann-von-Helmholtz-Platz 1, 76344 Eggenstein-Leopoldshafen, Germany; Tel.: +49 721 608 28970; Fax: +49 721 608 26368

Comparison between ACOM- and DF-TEM evaluation of grain growth and twin activity

As DF-TEM is commonly used to characterize the microstructure of nc materials, we performed a comparison between DF-TEM and ACOM-TEM. In Figure S1, DF-TEM images of sample set ncPd 2 are shown at different deformation states together with selected area electron diffraction (SAED). Figure S2 displays the grain size and the twin boundaries/grain for both the DF-TEM images evaluated by human inspection and the automatically evaluated ACOM-TEM images. As the twin density measured by DF-TEM is very low compared to ACOM-TEM, we assume that we mostly detect complete grains by DF-TEM and only a limited number of individual crystallites. Therefore, we compared the DF-TEM analysis with the ACOM-TEM derived grain size. To compare the twinning behavior we choose twin boundaries/grain as a metric for the ACOM-TEM, as this is the closest to the DF-TEM twin evaluation. For the grain size, we observe similar trends by DF-TEM and ACOM-TEM. However, the absolute values differ slightly as the human eye filter selects preferably bigger grains, whereas noise in ACOM-TEM images might create too many small grains. More significant is the difference for the twin boundaries/grain. While we detected only 0.005 twin boundaries/grain by DF-TEM, ACOM-TEM suggests a more realistic value of 1.1 for the twin boundaries per grain for sample ncPd 2. This huge difference in directly observed twins is mainly due to the ratio of the size of the diffraction aperture and the reciprocal space distance between diffraction spots of a set of twins or in other words, the likelihood of the reflection of one twin being within the objective aperture while the reflection for the other twin is blocked by the

aperture. Nevertheless, DF-TEM was able to detect the increasing twin density, which was much more pronounced by ACOM-TEM.

The advantages of ACOM-TEM are:

- The majority of all grains in the area of interest are taken into consideration,
- it is an user independent evaluation method,
- all in the 2D projection distinguishable twin boundaries are detected within the area of interest,

as long as the TEM sample is sufficiently thin compared to the thickness of the grains. These advantages result in a better statistical evaluation of the microstructure compared to DF-TEM. Moreover ACOM-TEM enables further evaluation such as texture analysis.

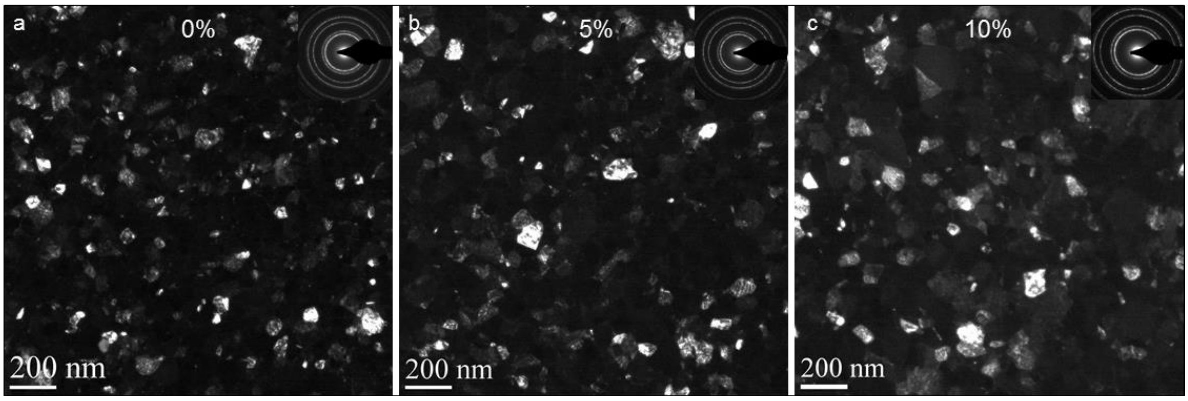

Figure S1: DFTEM images and selected area electron diffraction patterns (SAED) insets for the a) initial structure and after straining to b) 5% and c) 10%.

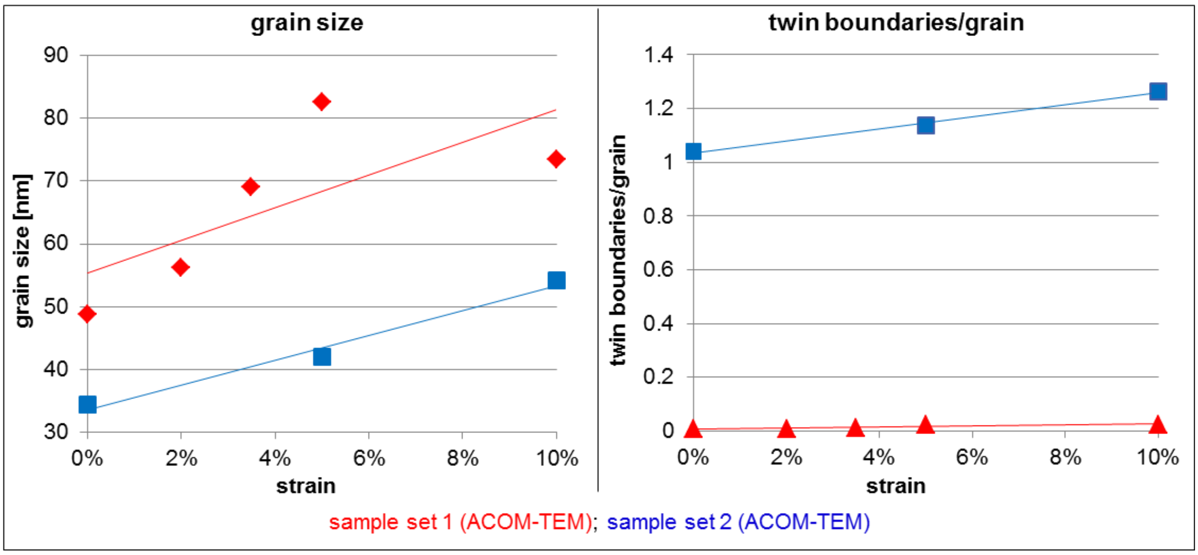

Figure S2: Comparison between DFTEM (red) and ACOM-TEM (blue) evaluation: a) Grain size and b) twin boundaries/grain as a function of strain.
